# Supplementary figures and images for: In the social amoeba Dictyostelium discoideum, shortened stalks may limit obligate cheater success even when exploitable partners are available
Source: PeerJ. 2024 Mar 29;12:e17118. doi: 10.7717/peerj.17118 (PMC10984163; doi:10.7717/peerj.17118)

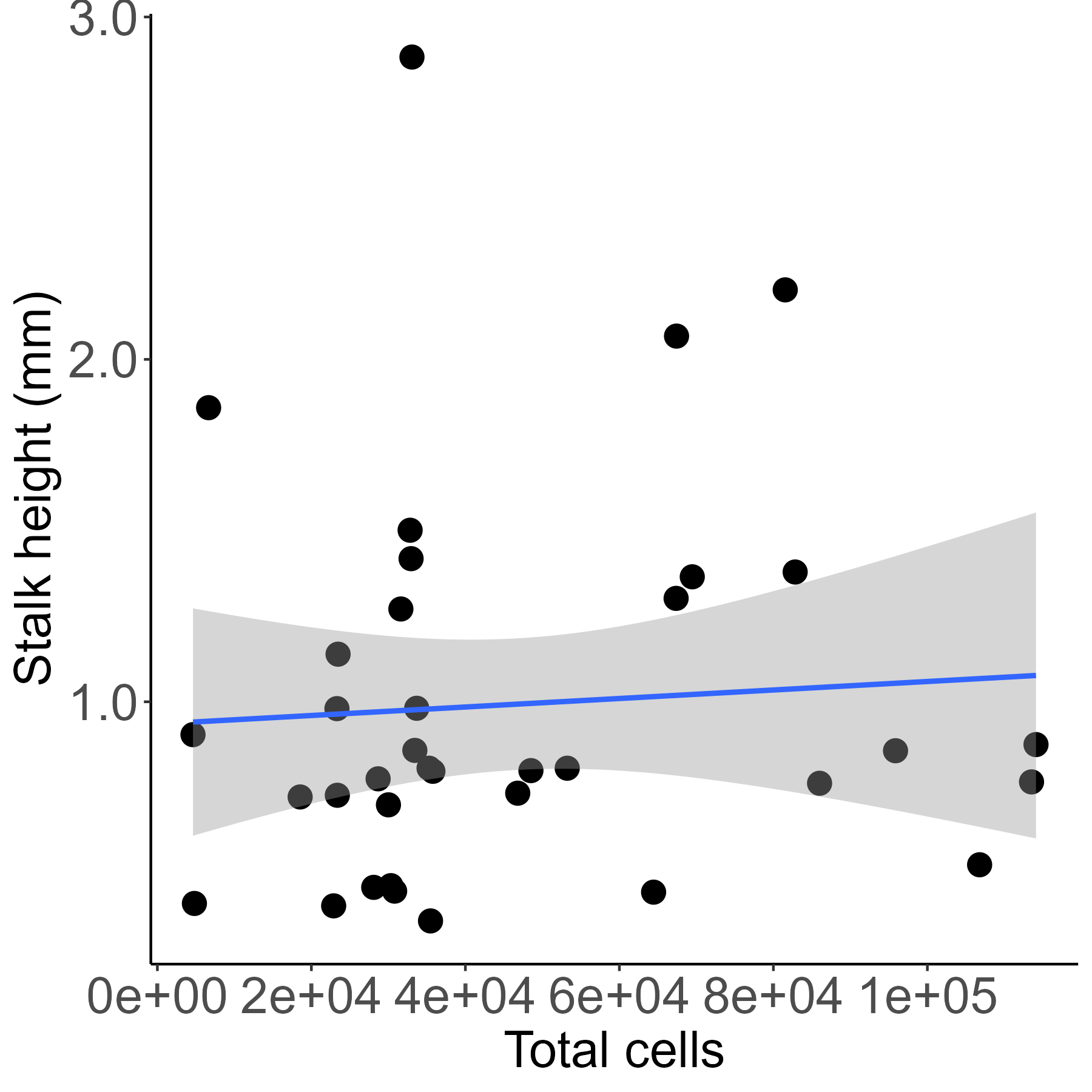

Supplement: Supplemental Information 1 — Each point represents measurement of a single fruiting body. Cell number does not predict stalk height (linear model, DF=34, t=0.391, p=0.698). Regression line is y=(1.242e-06)x + 0.935. R-squared = 0.0045. Shaded area is 95% CI. [file peerj-12-17118-s001.png]
